# Evaluation of real-time LAMP assay for detection of *Entamoeba* species in clinical stool samples from a tertiary care center in North India

**DOI:** 10.3389/fpara.2026.1937802

**Published:** 2026-10-01

**Authors:** Devyani Sharma, Usha Dutta, Divya Rattan, Upninder Kaur, Shinichi Inoue, Shinjiro Hamano, Ruby Jain, Kawaljeet Singh Sandha, Rakesh Sehgal, P. V. M. Lakshmi

**Affiliations:** 1 Department of Medical Parasitology, Post Graduate Institute of Medical Education and Research, Chandigarh, India; 2 Department of Gastroenterology, Post Graduate Institute of Medical Education and Research, Chandigarh, India; 3 Amity School of Biological Sciences, Amity University Punjab, SAS Nagar, India; 4 Department of Immunology, Nagasaki University, Nagasaki, Japan; 5 Department of Parasitology, Institute of Tropical Medicine (NEKKEN), Nagasaki University, Nagasaki, Japan & The Joint Usage/Research Center on Tropical Disease, Institute of Tropical Medicine (NEKKEN), Nagasaki University, Nagasaki, Japan; 6 Department of Microbiology, Civil Hospital, Manimajra, India; 7 Department of Microbiology, Aarupadai Veedu Medical College and Hospital, Pondicherry, India; 8 Department of Public Health and Community Medicine, PGIMER, Chandigarh, India

**Keywords:** *Entamoeba histolytica*, inflammatory bowel disease, molecular diagnostics, real-time LAMP, ulcerative colitis

## Abstract

**Background:**

Enteric protozoa infections contribute to diarrheal diseases in low and middle income countries, affecting those with limited access to clean water and sanitation. *Entamoeba histolytica* is an invasive water and food-borne parasite, associated with mucosal inflammation causing symptoms of amoebic colitis that can overlap with ulcerative colitis (UC). As conventional diagnostic methods such as microscopy cannot differentiate morphologically similar species, diagnostic challenges frequently arise. The present prospective study evaluated the clinical diagnostic performance of Real-Time Loop Mediated Isothermal Amplification Assay (LAMP) for detecting and differentiating *Entamoeba species* in clinical endemic settings, with a focus on patients with UC.

**Methods:**

The study was conducted at a tertiary care hospital in North India from January 2021 to January 2023. Stool samples (n=494) were collected across four groups: patients with UC (n=117), non-inflammatory bowel disease (IBD) diarrhea patients (n=177), community participants (n=100), and diseased controls (n=100). All samples were analyzed using microscopy, conventional PCR, real-time PCR, and real-time LAMP, followed by phylogenetic analysis for species confirmation. For real-time LAMP, the SREHP gene and LSU rRNA gene reactions were performed separately in single run to ensure accurate identification. Statistical evaluation was performed using Bayesian latent class analysis.

**Results:**

The sensitivity of microscopy, conventional PCR, real-time PCR, and real-time LAMP was calculated to be 39.4% 84.3%, 99.3%, and 99.3%, respectively, with 99.4–100% specificity for real-time LAMP. Five samples missed by conventional PCR were successfully detected by both real-time PCR and real-time LAMP. The overall infection rate across was 6.7% (33/494), with differential detection of Entamoeba species. Samples positive for *E. histolytica* showed amplification of both SREHP and LSU rRNA genes, whereas *E. dispar* and *E.moshkovskii* showed amplification of the LSU rRNA gene only. Among patients with UC, those with active disease had a higher incidence of *E. histolytica* infection (13/88) than those in remission (4/29).

**Interpretation:**

Real-time LAMP demonstrated specificity and sensitivity of 99.3% (92.5–100) equivalent to that of real-time PCR with an added advantage of shorter turnaround time than conventional PCR and real-time PCR (60 minutes vs. 200 minutes vs 120 minutes), facilitating prompt differential diagnosis of Entamoeba species in patients with UC. These finding support considering molecular tests in microscopy-negative cases to improve detection and differentiation of Entamoeba species in high-incidence areas and among high-risk patients. However, cost effectiveness and downstream impact on clinical outcomes warrant further study.

## 1 Introduction

*Entamoeba histolytica*, an invasive protozoan parasite is a significant contributor to global mortality associated with parasitic diseases (Moran et al., 2023; Sharma et al., 2025). Transmission in developing nations primarily occurs via the fecal-oral route whereas sexual contact among homosexual men and immigrants represent the main route in developed nations (Chou and Austin, 2020). In India, its prevalence ranges from 3% to 23%, particularly among children aged 5-12 (Gupta et al., 2022). *E. histolytica* has also been associated with certain water-borne outbreaks (Chen et al., 2001; Karanis et al., 2006). Clinical manifestations of amoebiasis vary from asymptomatic infection to severe complications, including extra-intestinal abscesses, perforating complications, toxic megacolon, and others, underscoring the need for early and accurate diagnosis (Morán et al., 2023).

While the *Entamoeba* genus comprises numerous commensal species, recent studies suggest that *E. moshkovskii* may also induce gastrointestinal symptoms (Sardar et al., 2023). Accurate species differentiation is critical for appropriate patient management, as *E. histolytica* requires anti-amoebic therapy (Morán et al., 2023).

Current diagnostic approaches encompass microscopy, serology, antigen & antibody detection, molecular techniques, and histology from colonoscopy specimens. Microscopy remains the most commonly used method but has significant limitations: poor sensitivity, dependence on microscopist expertise, and inability to differentiate morphologically similar species (Morán et al., 2023). Antigen tests may show cross-reactivity with other *Entamoeba* species such as *E. dispar*, while antibody tests have limited utility in endemic areas. Molecular techniques provide precise identification but typically require trained personnel and expensive equipment (Morán et al., 2023). Diagnostic tools that are rapid, affordable and deployable in low-resource settings are essential to achieve the United Nations sustainable development goals by enabling early case detection, targeted treatment, and outbreak control in endemic regions (Boehme et al., 2021; Yimer et al., 2024).

Loop-Mediated Isothermal Amplification Assay (LAMP), based on strand displacement synthesis carried out by Bst DNA polymerase using outer and inner primers, has garnered attention for its efficacy in detecting *E. histolytica* and distinguishing it from non-pathogenic *Entamoeba* species (Foo et al., 2017). The reaction can be performed using simple equipment such as a water bath, heat block, or a portable fluorometer (Njiru, 2012). Real-time LAMP offers advantages over conventional LAMP methods especially in tertiary care hospitals which usually have high patient load. The minimal interference from non-target DNA, capability for multi- target detection with differentiation based on melt curve analysis and the closed tube format reduces the risk of aerosol contamination. Whereas, the minimal post amplification processing also makes it suitable for routine use in busy and resource-limited laboratory settings (Moehling et al., 2021; Crego-Vicente et al., 2024). Although multiplex real-time LAMP allows multi-target detection via melt-curve analysis, but in the present study, target differentiation was limited because the master mix relies on a single double strand intercalating dye (Ampligene India Pvt. Ltd., Ahmedabad, India) (Moehling et al., 2021; Crego-Vicente et al., 2024). The annealing temperatures of the SREHP and LSU rRNA gene amplicons were close, making clear differentiation difficult in the duplex format. Clinical samples were therefore evaluated as a parallel monoplex assay (two tubes per sample) in a single run, but this was found to increase the reagent costs, which requires further cost evaluation in clinical setting.

The rapid change of lifestyle is leading to an increased incidence of ulcerative colitis (UC) in developing nations, which already bear a significant burden of endemic infections. Therefore, individuals with UC experiencing diarrhea exacerbations should be evaluated for concurrent infections before escalating immunosuppressive therapy as doing so without addressing an underlying infection can be counterproductive (Shirley and Moonah, 2016; Zhu et al., 2023). Various gastrointestinal infections have been documented during flares of inflammatory bowel disease (IBD), primarily those caused by bacteria. However, limited research exists on the diagnosis and effects of parasitic infections like *E. histolytica* during these flare-ups, an area that has largely been neglected until now (Babić et al., 2016; Limsrivilai et al., 2020; Hamed et al., 2023; Soylu et al., 2025).

The role of real-time LAMP in detecting *Entamoeba* infections in clinical practice in India, particularly in the IBD context remains largely unexplored. This study primarily aimed to evaluate the diagnostic performance of real-time LAMP compared to microscopy and conventional molecular methods, and secondarily to determine the prevalence of *Entamoeba* species in UC patients and other cohorts.

## 2 Materials and methods

### 2.1 Study setting and study design

The study was carried out in a tertiary care center (North India) from January 2021 to January 2023 after ethical approval from the Institutional Ethics Committee (INT/IEC/2022/SPL-766) and followed the ethical norms of the Helsinki Declaration (2013) and Indian Council of Medical Research Guidelines (2019) (World Medical Association, 2013; Behera et al., 2019). Written informed consent was obtained from all participants before enrollment.

### 2.2 Study subjects

The study included patients aged >18 years who sought medical attention in the Gastroenterology Outpatient Department (OPD) and Emergency Medical OPD of Nehru Hospital at PGIMER. Study participants were categorized into four groups (**Figure 1**): UC patients (n=117): Diagnosed according to standard clinical, endoscopic, and histological criteria in accordance with European Crohn’s and Colitis Organization guidelines. Disease activity was classified as active (mild, moderate, or severe) or in remission based on clinical and endoscopic assessment (Raine et al., 2021).

**Figure 1:**
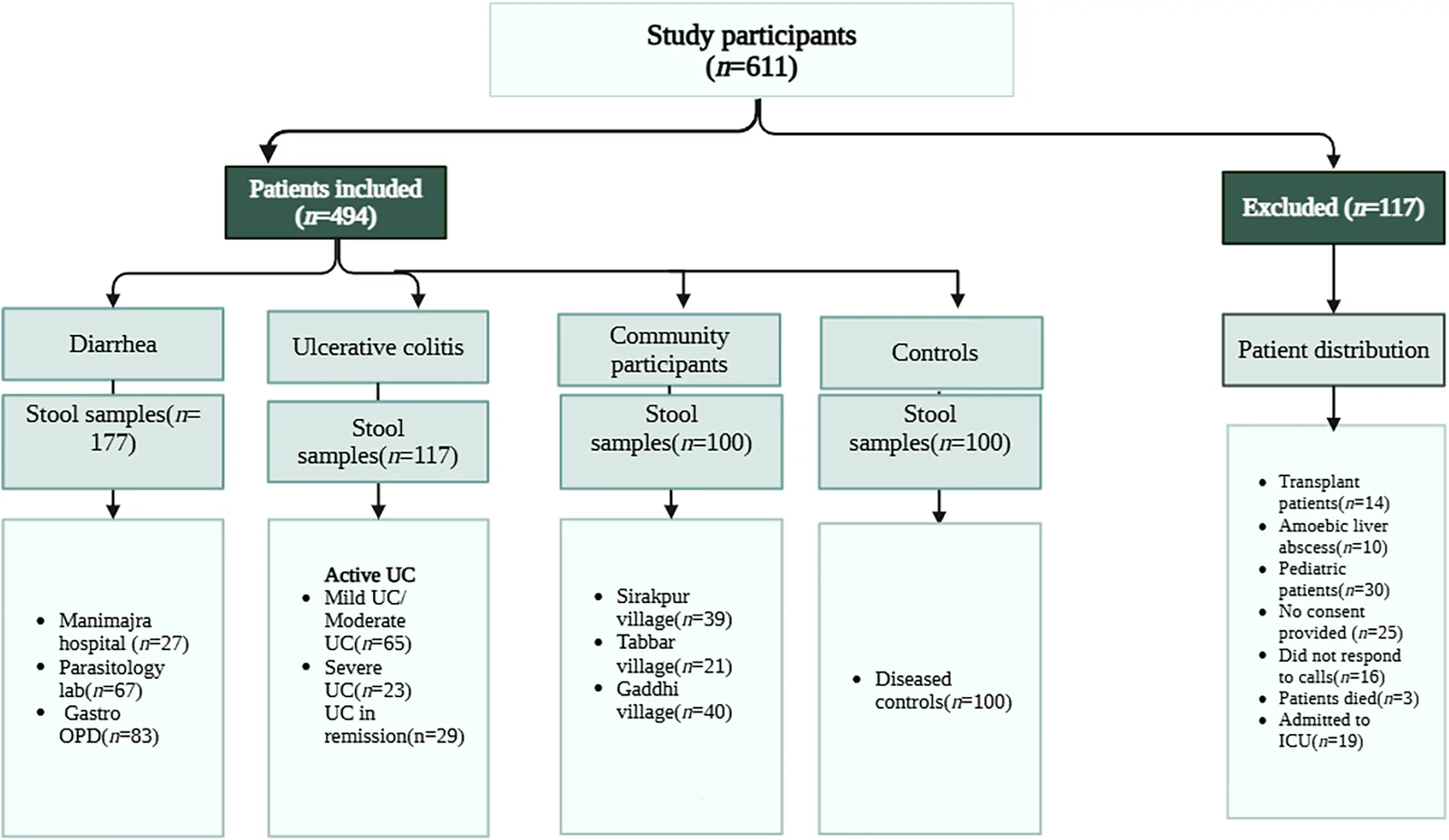
Distribution of study participants (https://www.biorender.com/).

Non-IBD diarrhea patients (n=177): Persistent diarrhea (liquid stool consistency for 2–3 weeks) or chronic diarrhea (> 4 weeks), without IBD diagnosis (Arasaradnam et al., 2018).

Community controls (n=100): Individuals recruited from rural communities (villages of Gaddhi, Tabbar, and Sirakpur).

Diseased controls (n=100): Patients with gastrointestinal symptoms other than diarrhea. The referenced formula was used for sample size determination (Pourhoseingholi et al., 2013).

### 2.3 Storage and processing of samples

Stool samples were collected in sterile Uricols containers (HiMedia^®^) and transported to the Medical Parasitology laboratory for processing.

### 2.4 Diagnostic techniques

#### 2.4.1 Microscopic examination

The stool samples were processed using Faecal Parasite concentrator (Mini Parasep^®^ SF, catalogue 108920) and examined under a light microscope at both low and high magnifications, the objective lens used was 10x and 40x. For quality control a known negative fecal specimen was processed alongside patient samples (Mewara et al., 2019; Khurana et al., 2023).

#### 2.4.2 Optimization of DNA extraction

The extraction was done using QIAmp^®^ Fast DNA Mini Stool Kit (QIAGEN, Hilden, Germany/Catalogue no. 51604) following the manufacturer’s instructions with slight modifications to improve yield from fecal samples. The stool samples were washed twice with phosphate-buffered saline (PBS) prior to extraction. The first incubation period was extended to 50 minutes and the second to 45 minutes. The samples were vortexed vigorously for 10 minutes in two 5 minutes cycles before and after each incubation. Proteinase K volume was increased to 25 µL, and elution was performed with 50 µL instead of 200 µL. The concentration and purity of extracted DNA were quantified using a Nanodrop One/OneC Microvolume UV-Vis Spectrophotometer (Thermo Scientific, Waltham, Massachusetts).

### 2.5 Molecular techniques

#### 2.5.1 Housekeeping gene

All samples were first subjected to conventional PCR targeting the human Glyceraldehyde-3-phosphate dehydrogenase (*GAPDH*) housekeeping gene to confirm DNA quality; it was not intended as a dedicated inhibition control (Barber et al., 2005). The primers, reaction volume and amplification conditions are mentioned in **Table 1**.

**Table 1:** Details of the oligonucleotides used in the current study.

Technique	Gene	Forward (5’-3’) Reverse (5’-3’)	Reaction volume	Amplification conditions	Product size (bp)	Reference
Conventional PCR (Housekeeping gene)	*GAPDH* *(Glyceraldehyde-3-phosphate dehydrogenase)*	F-CCAAAGTCATCCATGACAACTTTGGT R-TGTYGAAGTCAGAGGAGACCACCTG Y=C/T	Total reaction volume - 25 µL Go Taq Green master mix (2X conc.) -12.5µL, Forward and reverse primers (10 µM conc.) 0.5 µL, nuclease-free water – 9.5 µL, DNA template (2 µL).	Denaturation: 94 °C for 5 minutes, 30 cycles of annealing at 59 °C for 30 seconds followed by extension at 72 °C for 1 minute.	380bp	(Barber et al., 2005)
Conventional PCR (Entamoeba species)	*SSU rRNA*	*E. histolytica* (Forward is same for all three species) F-ATGCACGAGAGCGAAAGCAT R-GATCTAGAAACAATGCTTCTCCTCT *E.dispar* R-CACCACTTACTATCCCTACC *E.moshkovskii* R-TGACCGGAGCCAGAGACAT	Total reaction volume -25 µL Go Taq Green master mix (2X conc.) -12.5 µL, Forward and reverse primers (10µM conc.)- 0.5 µL, Nuclease free water -9.5µL, DNA template (2 µL).	Initial denaturation step at 94 °C for 5 minutes, denaturation at 94˚C for 30 seconds, followed by 30 cycles of annealing at 58˚C for 30 seconds, and extension at 72˚C for 1 minute.	166bp (Eh) 752bp (Ed) 580bp (Em)	(Hamzah et al., 2006)
Real-time PCR	*SSU rRNA*	*E. histolytica* F-AAGCATTGTTTCTAGAT CTGAG R-AAGAGGTCTAACCGAAATTAG *E.dispar* F-TCTAATTTCGATTAGAACTCT R-TCCCTACCTATTAGACATAGC *E.moshkovskii* F-GAAACCAAGAGTTTCACAAC R-CAATATAAGGCTTGGATGAT	Total reaction volume -25 µL SYBR Green^®^Premix Ex Taq™ II master mix 12.5 µL, Forward and reverse primers (10µM conc.)- 0.5 µL, Nuclease free water -9.5 µL, DNA template (2 µL).	Initial heating step at 95 °C for five minutes, denaturation at 95 °C for 15 seconds then 35 cycles of annealing at 60 °C for 30 seconds; melt curve analysis (MCA) was conducted by gradually raising the temperature from 65 °C to 95 °C in order to keep a check for the formation of non-specific products such as primer dimers.	–	(Dinoop et al., 2016)
Real-time LAMP	*SREHP(E. histolytica)* *LSU rRNA (All 3 Entamoeba species)*	*SREHP gene* FIP (Forward inner primer) GCTTCGTTCTTTAAAAATACACCGTCATTCTTGATTTGGATCAAGAAGT BIP (Backward inner primer) AGTAGCTCAGCAAAACCAGAATCAC TTGCTTTTTCATCTTCATCA F3 (Forward outer primer) TGCATTCACTAGTGCAACT B3 (Backward outer primer) GCTTGATTCTGAGTTATCACTTG LB (Loop backward primer) AAGTTCAAATGAAGATAATGAA *LSU rRNA gene* FIP (Backward inner primer) TACGCCATTTCGTTTCCTTACTCGATTT CTTAACTGATACTCGACCG BIP (Backward inner primer) AGATTGAAACTGTCCTTAGTGCAGCAGT TCTAAGATGTTTTTTTCCTC F3 (Forward outer primer) CCTGAAAATGGATGGCATTA B3(Backward outer primer) CCCTAATCCAAGTAATGTTGTT LB (Loop backward primer) CTTGGTGGTAGTAGCAAATACTAAG	The total reaction volume was 25 µL, consisting of 12.5 µL of Isothermal master mix, 2.5 µL of primer mix and 1 µL of template and 9 µL distilled water.	The mixture was run at 63 °C for 60 minutes and termination at 80 °C for 5 minutes.	Samples positive for *E. histolytica* exhibited amplification in both genes (*SREHP* and *LSUrRNA*) and samples positive for *E. dispar* and *E. moshkovskii* showed amplification in only one gene (*LSU rRNA)*.	(Foo et al., 2017)

#### 2.5.2 Conventional PCR and real-time PCR

Differential detection of *Entamoeba* species was carried out via conventional PCR, real-time PCR, and real-time LAMP (Hamzah et al., 2006; Dinoop et al., 2016; Foo et al., 2017). The *E. histolytica* axenic strain HM1: IMSS and *E. dispar* isolated from stool culture were used as positive control (Clark et al., 1991), which were already maintained in the Department of Medical Parasitology, PGIMER (Clark and Diamond, 2002). *E. moshkovskii* (Laredo strain) was also used as a positive control (Clark et al., 1991).

*SSU rRNA* gene amplification was performed using Bio-Rad C1000 Touch™ thermal cycler. Band size of specific PCR products was confirmed by gel electrophoresis using ethidium bromide dye and visualized using a gel documentation system (Protein Simple Alpha Mager System Auto Mated Gel Imaging, U.S.A).

Real-time PCR was run on CFX Touch 96-well Light Cycler real-time PCR detection system (Bio-Rad, USA) using SYBR Green as the detection dye. For clear identification, each sample was run three times using three distinct primer sets unique to the three species (*E. histolytica, E. dispar*, and *E.moshkovskii*).The reaction mixture included a master mix composed of 12.5 µL of TB SYBR Green^®^Premix Ex TaqTM II master mix (TliRNase H plus, Takara Bio, New Delhi, India) 0.5 µL each of forward and reverse primers, 9.5 µL nuclease-free water and 2 µL template DNA (at a concentration of 50 ng/µL for all samples). The reaction was set up in opaque real-time PCR strips with a volume of 0.2 ml. Melt curve analysis (MCA) was conducted by gradually raising the temperature from 65 °C to 95 °C in order to check for the formation of primer dimers. The obtained results were then analyzed using the Bio-Rad CFX Manager Software.

#### 2.5.3 Real-time LAMP assay

Real-time LAMP was performed via targeting two genes: serine-rich protein (*SREHP*) specific for *E. histolytica* and large subunit ribosomal RNA *(LSU rRNA*) common to all three species, allowing detection and differentiation of the species (Foo et al., 2017). The reaction was carried out in a transportable real-time fluorometer (Optigene III, UK, https://www.optigene.co.uk/instruments/genie-iii/) The ratio of outer primer to inner primer for E1 was 1:12,and for E2 it was 1:3. The final concentration of the primers for the RT- LAMP is mentioned in **Table 2** (Foo et al., 2017). The total reaction volume was 25 µL, consisting of 12.5 µL of Isothermal master mix (Ampligene India Pvt. Ltd., Ahmedabad, India), 2.5 µL of primer mix and 1 µL of template and 9 µL distilled water. The isothermal master mix utilized in the study contained a double stranded DNA binding intercalating dye and contamination was minimized by not opening tubes after amplification, using aerosol barrier tips and including negative controls.

**Table 2:** Final concentration of the primers for the LAMP.

Gene	Forward inner primer (FIP)	Backward inner primer (BIP)	Forward outer primer(F3)	Backward outer primer(B3)	Loop primer (LP)
E1(*SREHP)*	2 µM	2 µM	0.167µM	0.167µM	0.67µM
E2(*LSU rRNA)*	0.5µM	0.5µM	0.167µM	0.167µM	0.67µM

#### 2.5.4 Limit of detection and specificity of molecular techniques

The limit of detection was determined using serial dilutions of *E. histolytica* HM1: IMSS DNA ranging from 1nanogram (ng) to 10 femtogram (fg). The stool samples were spiked and used to determine the least amount of DNA that could be detected with molecular techniques (Blessmann et al., 2002).

#### 2.5.5 Phylogenetic analysis

Out of 33 positive samples only 10 representative samples were subjected to QIA quick PCR Purification Kit (Cat. No. 28104, Qiagen, Hilden, Germany) and Sanger sequencing using an ABI 3730 DNA Analyzer at the Central Sophisticated Instrumentation Cell (CSIC), PGIMER. The 10 samples were selected based on the intensity of bands as seen on conventional PCR gel electrophoresis representing the three species. Sequences were analyzed using Finch TV software (https://digitalworldbiology.com/FinchTV) and compared with reference sequences in NCBI GenBank using BLAST after which phylogenetic analysis was conducted (Malaa et al., 2019).

#### 2.5.6 Statistical analysis

Bayesian latent class analysis was performed using Model 105 (http://mice.tropmedres.ac/home.aspx) to evaluate the sensitivity, specificity, positive predictive value (PPV), and negative predictive value (NPV) of microscopy, conventional PCR, real-time PCR, and real-time LAMP (Lim et al., 2013). Statistical Guidance on Reporting Results from studies Evaluation Diagnostic tests (**Supplementary Data Sheet**) was also assessed for diagnostic tests (Bossuyt et al). In the context of the current study, microscopy cannot differentiate among morphologically similar species and PCR is only applicable in well-established setups, therefore Bayesian latent class analysis was used.

## 3 Results

### 3.1 Study population characteristics of patients with ulcerative colitis

A total of 611 stool samples were collected; of these,117 were excluded (**Figure 1**), and the final analysis was carried out on 494 participants (mean age: 37.5 ± 14.2 years; males: 68.4%, females: 31.6%). Among UC patients (n=117), the most common disease phenotype was left-sided colitis (E2, 37.9%), followed by pancolitis (E3, 14.8%) and proctitis (E1,12.8%). Complete data could not be obtained for 20.5% of patients with UC and no contact could be established with 14% of the patients. The disease duration exceeded two years in 57.9% of UC patients. Seventy-nine patients (67.5%) experienced a relapsing-remitting course, while the remainder had a first or single episode. Extra-intestinal manifestations were present in 14 patients (12.0%), with arthritis/arthralgia being most common (n=13) and one case of pyoderma gangrenosum.

### 3.2 Microscopic examination

Microscopic examination identified *Entamoeba histolytica/dispar/moshkovskii* cysts or trophozoites in 15 samples. All microscopy positive samples were confirmed by molecular methods except two, and it missed 20 of the 33 molecularly confirmed positive cases, demonstrating its limited sensitivity in this setting.

### 3.3 Molecular techniques

All the samples exhibited amplification of the *GAPDH* housekeeping gene (**Figure 2.1**). Out of the 15 microscopic positive samples, all the molecular techniques confirmed 13 samples except 2, with an additional twenty positive samples. In total 28 samples were positive by conventional PCR, 33 by real-time PCR and real-time LAMP (**Figures 2**, **3**). For real-time-LAMP, samples positive for *E. histolytica* exhibited amplification in both genes (*SREHP* and *LSUrRNA*) while samples positive for *E. dispar* and *E. moshkovskii* showed amplification in only one gene (*LSU rRNA*) (**Figure 3**).

**Figure 2:**
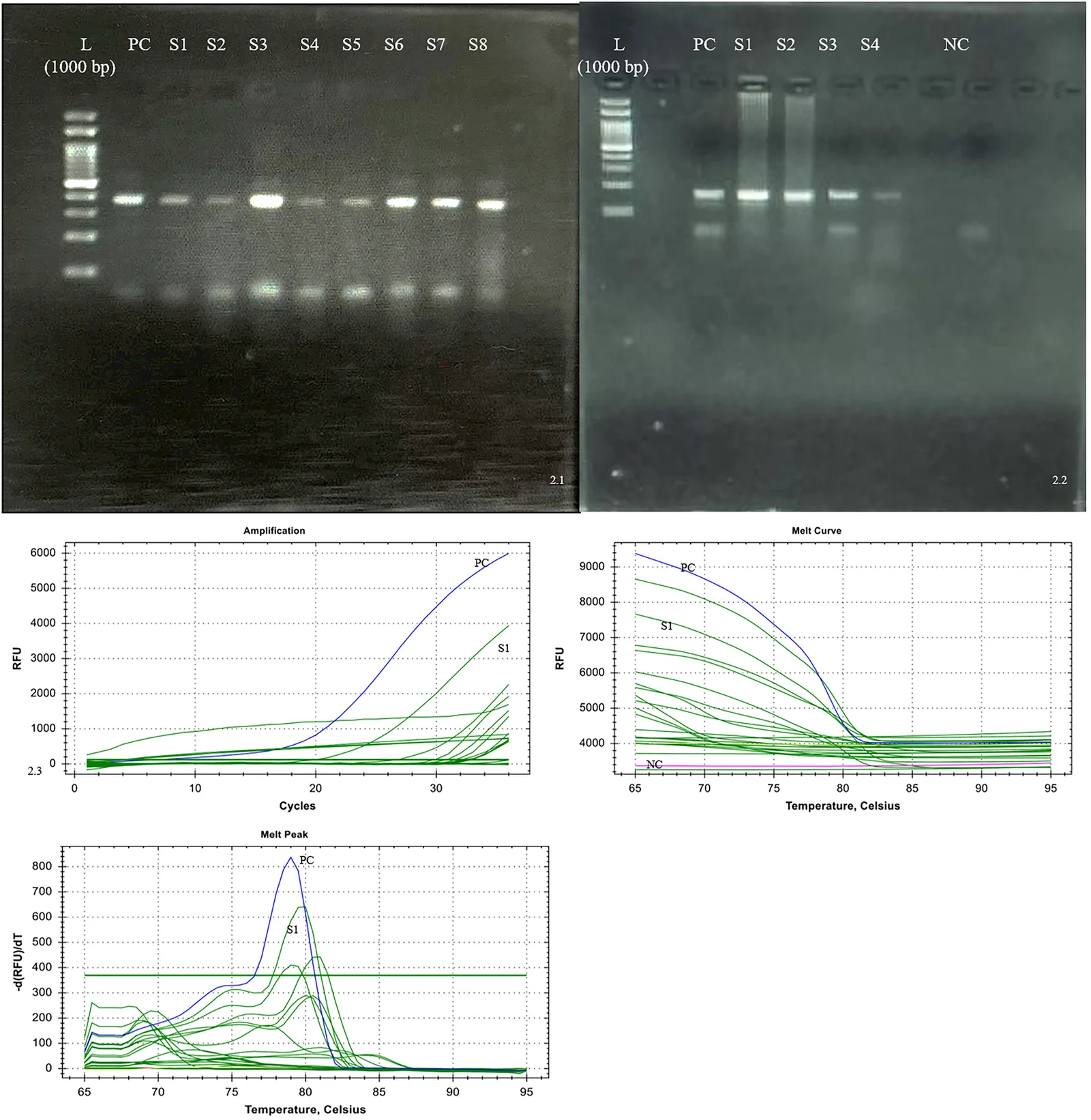
Representative agarose gel image (Ladder-L (1000 bp), Negative control (NC), Positive control (PC), Stool samples (S1). **(1)** *GAPDH* housekeeping gene (380 bp; 1.8% gel). **(2)** Conventional PCR -*SSU rRNA* gene (*E. histolytica* -166 bp) 1.8% gel. **(3)** Representative pictures of Real-Time PCR for *SSU rRNA* gene of *Entamoeba* species: Negative Control (NC), Positive control (PC), Stool sample (S1), Amplification curve, Melt curve analysis image of positive and negative stool samples for *Entamoeba* species.

**Figure 3:**
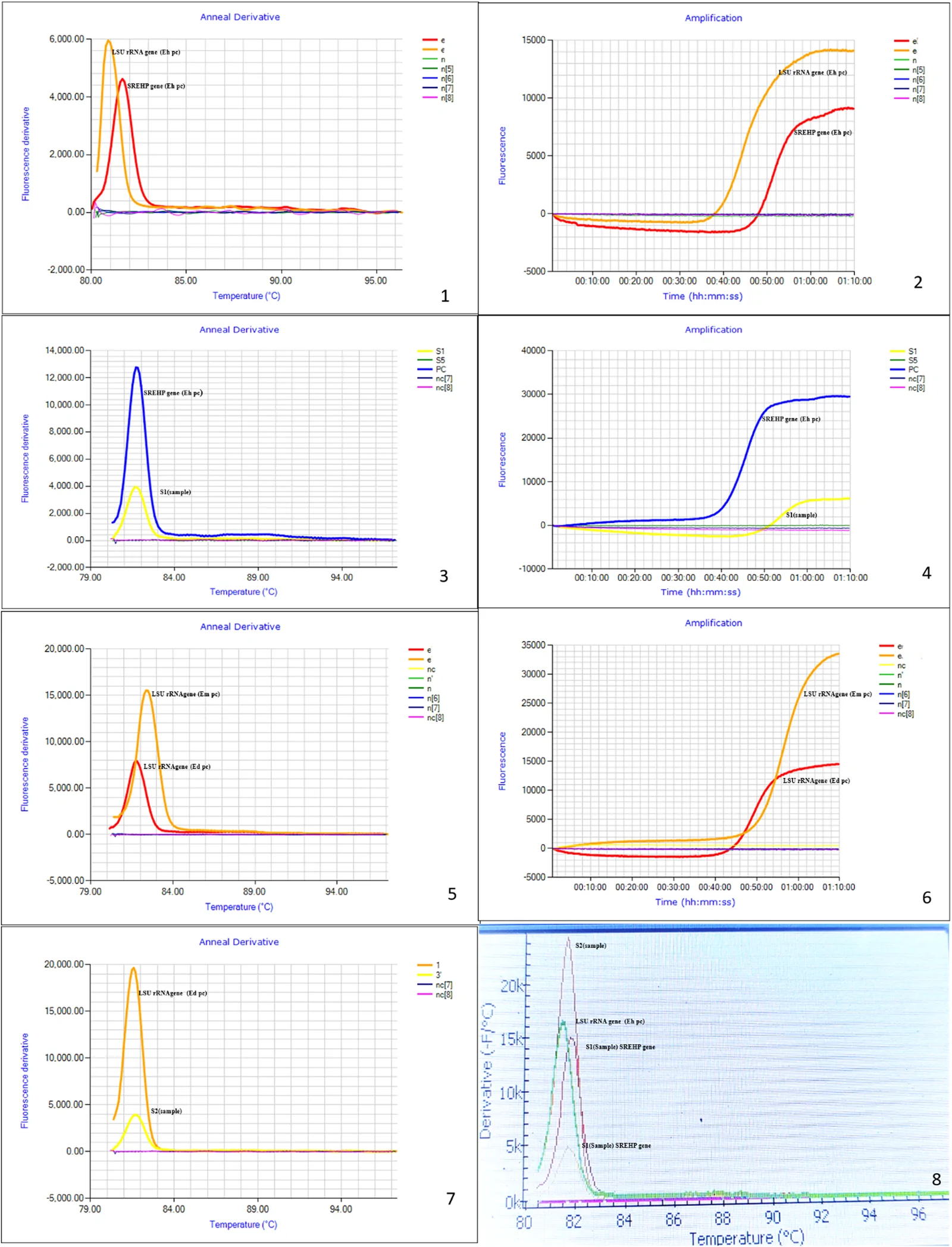
Representative Real-Time LAMP assay anneal derivative and corresponding amplification curve for *Entamoeba* species identification. **(1, 2)** *E. histolytica* positive control showing melting peaks for the *SREHP* gene (81.1°C) and the LSU *rRNA* gene (81.7°C). **(3, 4)** Detection of *E.histolytica* in clinical sample S1, matehing the positive control peak at 81.7°C (*SREHP* gene). **(5, 6)** Comparative positive controls for the *LSU rRNA* gene, differentiating *E. moshkovskii* (82.0°C) from *E.dispar* (81.9°C). **(7)** Detection of *E.dispar* in clinical sample S2, aligning with the positive control peak at 81.9°C (*LSU rRNA* gene). **(8)** *E. histolytica* positive sample exhibiting amplification in both *SREHP* and *LSU FRNA*. The primer sets were evaluated in separate reaction tubes for clinical screening in order to prevent overlapping of closely spaced anneal derivative peaks, ensuring clear species differentiation. (PC, Positive Cogtrol; NC, Negative Control; S1/S2/N, clinical samples).

In the UC group (n =117), 88 patients had active disease (twenty-three cases of severe UC and sixty-five cases of mild to moderate UC) and twenty-nine patients were in remission. Overall, twenty patients tested positive for Entamoeba infection: seventeen had *E. histolytica* and three had *E.dispar*. *E. histolytica* was detected in seventeen patients including 7/23 (severe UC), 6/65(mild to moderate UC) and 4/29 (remission cases). *E.dispar* was found in two remission cases and one moderate UC case.

In the non-IBD diarrhea group (n=177), eight patients tested positive: five for *E. histolytica* and three for *E. dispar*. Among asymptomatic community controls (n=100), two individuals tested positive for *E. dispar*, and one for *E. histolytica*. Diseased controls (n=100) showed one case of *E.dispar* infection and one of *E.moshkovskii*. Entamoeba species were detected in 33 stool samples by real-time LAMP, 23 of these samples were positive for *E. histolytica*, 9 for *E.dispar* and 1 had *E.moshkovskii* infection (**Table 3**). *E. histolytica* incidence was significantly higher in ulcerative colitis patients (both active and remission) than in controls (p<0.001), though rates did not differ significantly between active UC and remission cases (p=0.898).

**Table 3:** Detection rates of *Entamoeba* species by different diagnostic methods.

Method	Total positive	*E. histolytica*	*E. dispar*	*E. moshkovskii*
Microscopy	**15**	**ND**	**ND**	**ND**
Conventional PCR	**28**	19	08	01
Real-time PCR	**33**	23	09	01
Real-time LAMP	**33**	23	09	01

ND, Not differentiated by microscopy alone. Bold, Total number of positive samples by respective technique.

Initially, the LAMP assay was performed by adding both E1 and E2 primer sets in the same reaction using standard positive controls. Although amplification was observed for both targets, the annealing temperatures of the SREHP and LSU amplicons were close, making clear differentiation difficult in the duplex format. Therefore, for clinical samples, the E1 and E2 reactions were performed separately in the same run to ensure accurate identification.

For real-time LAMP*, E. histolytica* positive samples showed amplification of both genes, with results obtained as two fluorescent peaks (two separate monoplex tubes), while *E.dispar* and *E.moshkovskii* showed amplification of only *LSU rRNA* gene resulting in a single fluorescent peak. Real-time LAMP reduced the detection time to an hour compared to real-time PCR and conventional PCR. It was operated in a portable machine at an isothermal temperature and demonstrated the capability for multi-species detection (Optigene, UK, https://www.optigene.co.uk/instruments/genie-iii/). The limit of detection via serial dilution method was found to be 1 ng (nanogram)for conventional PCR, 1 pg (picogram) for real-time PCR and 10 fg (femtogram) for real-time LAMP. The highest sensitivity among the three molecular techniques was found to be that of real-time LAMP. **Table 4** summarizes the diagnostic characteristics, including sensitivity, specificity, positive predictive value (PPV) and negative predictive value (NPV) of the techniques.

**Table 4:** Diagnostic performance of detection methods using Bayesian latent class analysis.

Parameters [Bayesian latent class model (%)]				
Method	Sensitivity (%)	Specificity (%)	PPV (%)	NPV (%)
Microscopy	39.4 (24.0–56.0)	99.5 (98.6–99.9)	85.8 (63.5–97.1)	95.8 (93.8–97.4)
Conventional PCR	84.3 (69.8–94.1)	100 (99.5–100)	99.2 (91.3–100)	98.9 (97.7–99.6)
Real-time PCR	99.3 (92.5–100)	100 (100–100)	100 (100–100)	100 (99.5–100)
Real-time LAMP	99.3 (92.5–100)	100 (99.4–100)	99.3 (92.0–100)	100 (99.4–100)

Bayesian latent class model assumed that all tests evaluated are imperfect. Values shown are estimated median with 95% credible interval. PPV, positive predictive value; NPV, negative predictive value.

### 3.4 Phylogenetic analysis of the isolated *Entamoeba* species detected from stool samples

The *SSU rRNA* gene was sequenced in 10 stool samples positive for *Entamoeba* species and the sequences were deposited in GenBank. The analysis was performed using NCBI BLAST which indicated a high degree of sequence similarity (>95%) with other *Entamoeba* sequences in the database. The isolated strains clustered together with the reference sequences as depicted by the phylogenetic tree but low bootstrap values were also observed among the internal nodes (**Figure 4**).

**Figure 4:**
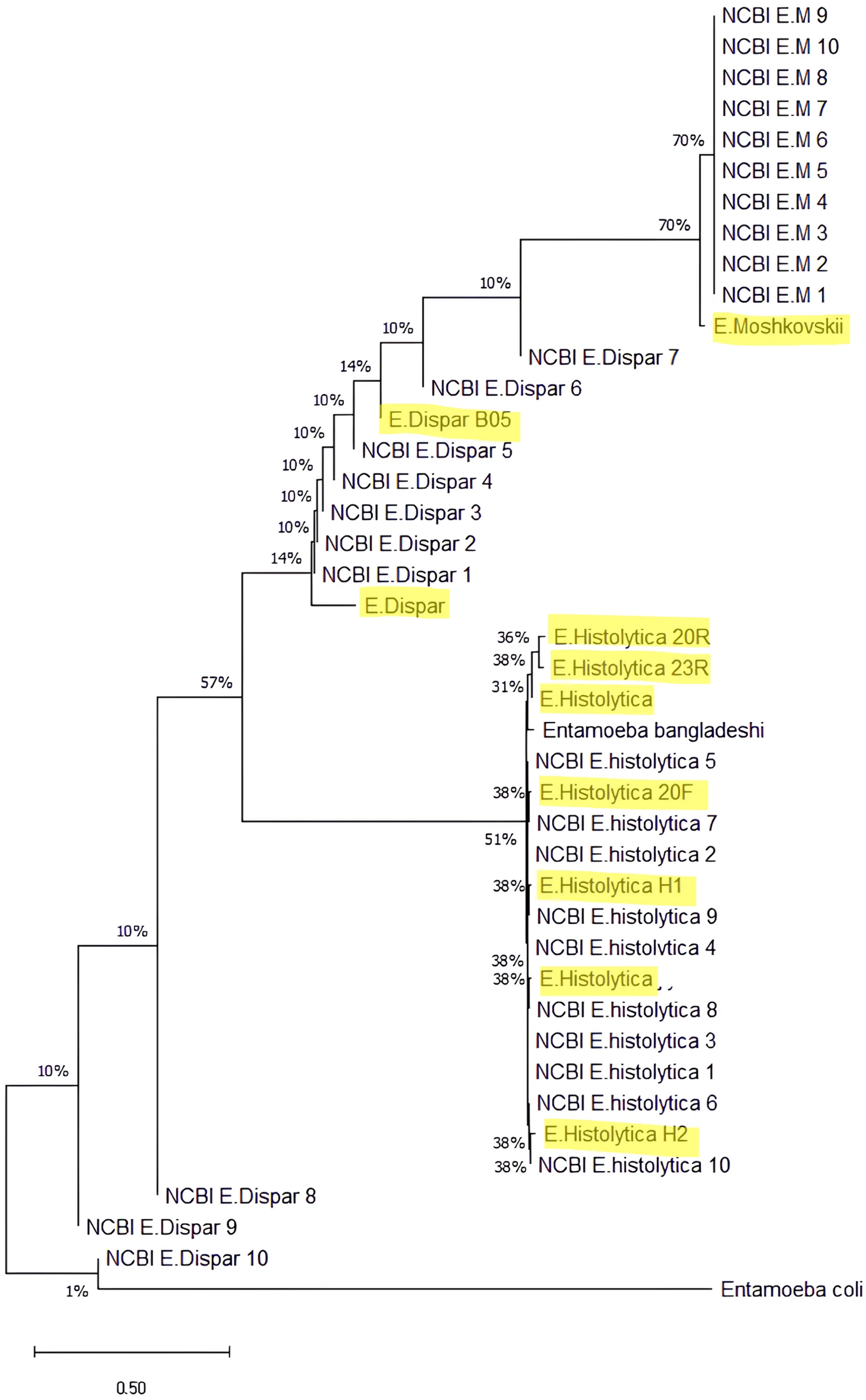
Molecular phylogenetic evaluation of Entamoeba isolates (SSU rRNA gene) by neighbour joining method. Yellow highlighted names represent the Entamoeba sequences in the current study whereas unmarked sequences are the similar sequences downloaded from GenBank database. Numbers at the nodes indicate bootstrap support values and the scale bar (0.50) represents the number of nucleotide substitutions per site.

## 4 Discussion

This study demonstrates that real-time LAMP is a sensitive (99.3%,92.5 - 100) and specific (100%,99.4-100) method for detecting and differentiating *Entamoeba* species in clinical samples, with performance equivalent to real-time PCR but with reduced turnaround time (60 minutes vs approximately 120 minutes for real-time PCR, and 200 minutes for conventional PCR). The differentiation of species addresses a diagnostic gap where microscopy alone is inadequate and is useful in resource-limited endemic settings (Nath et al., 2018; Sharma et al., 2026). Among UC patients, the prevalence of *E. histolytica* infection was notably elevated in the UC group, 14.8% (13/88) in active disease and 13.8% (4/29) in remission disease in comparison to other groups. Various studies have assessed the effectiveness of LAMP for identifying *Entamoeba* in fecal samples, and it has also been utilized for detecting other parasitic infections such as *Malaria, Leishmaniasis, Giardia* and *Cryptosporidium* in wastewater, *Strongyloides*, and *Schistosomiasis* (Fernández-Soto et al., 2016; Gallas-Lindemann et al., 2016; Lucchi et al., 2016; Nzelu et al., 2019; Diego et al., 2021; Mewara et al., 2023).

Previous studies have reported variable rates of amoebiasis in IBD populations, ranging from 2.5% to 38% depending on the geographical region and diagnostic methods (Babić et al., 2016; Limsrivilai et al., 2020; Hamed et al., 2023; Soylu et al., 2025). The findings align with other reports and underscore the importance of excluding parasitic infections before escalating immunosuppression (Gonzales et al., 2006; Shirley and Moonah, 2016; Soylu et al., 2025).

Microscopy, while widely available and inexpensive, demonstrated poor sensitivity 39.4% (24.0 – 56.0) in our study and cannot differentiate *E. histolytica* from non-pathogenic species. Moreover, there were two samples that were found to be positive by microscopy alone. Since microscopy has poor sensitivity and depends on microscopist expertise, this limitation results in both missed diagnosis and potential for overtreatment (Garcia et al., 2018; Morán et al., 2023). Conventional PCR improved sensitivity to 84.3% but missed 5 cases detected by real-time methods, likely due to PCR inhibitors in some samples (Ellison et al., 2006). Real-time PCR and real-time LAMP showed equivalent sensitivity with 100% specificity. Similar results have been obtained in the case of ALA patients (Khunger et al., 2024). The real-time LAMP assay offers several practical advantages: isothermal amplification eliminates the need for thermal cycling equipment, reducing infrastructure requirements and energy costs with rapid results in almost 60 minutes. The portable Genie III fluorometer operates on battery power, facilitating point of care testing in resource limited settings and visual color change in some LAMP formulations which allows result interpretation without instrumentation. However, the real-time fluorometricapproach used in this study provided quantitative data and melt curve analysis (Njiru, 2012; Moehling et al., 2021; Crego-Vicente et al., 2024). In the present study only 33 cases were positive, which resulted in a similar diagnostic sensitivity for real- time LAMP and real-time PCR, hence large multicentric studies are required to assess further diagnostic performance.

The relatively low infection rates observed in non-IBD diarrheal patients and community controls may be explained by several factors. Self-treatment for mild diarrheal episodes, improved hygiene practices in rural areas supported by initiatives such as Swachh Bharat Abhiyan (Dandabathula et al., 2019). The sampling was conducted shortly after the COVID-19 pandemic marked by movement restrictions and decreased consumption of outside food which likely reduced fecal-oral transmission. The study also excluded pediatric patients and amoebic liver abscess groups that typically show higher infection rates (Dinoop et al., 2016; Gupta et al., 2022). Moreover, only a single stool sample collection approach in these groups may have reduced diagnostic sensitivity compared to the standard three sample protocol; however, collecting multiple samples was not feasible within the study setting (Puebla et al., 2023).

The study has certain limitations; firstly, the sample size for patients with UC (n=117) was adequate for the primary objective, but it limited the ability to perform detailed subgroup analysis based on disease phenotype and treatment regimes. Since this cross-sectional study was primarily diagnostic in nature, it could not establish temporal relationships or causality between infection and disease activity. The effect of *E. histolytica* treatment on UC outcomes could not be assessed, and cost analysis could not be performed. For the clinical samples (real-time LAMP), the *SREHP* gene and *LSUrRNA* gene reactions were performed separately to ensure accurate identification, which also added an additional cost in this study and requires further evaluation of its clinical cost-effectiveness. One method to overcome this limitation is utilizing the instruments dual channel fluorescence system with sequence specific probes, enabling single tube multiplexing (Optigene III, UK, https://www.optigene.co.uk/instruments/genie-iii/). The specificity of the primers is based solely on the previously published validation by Foo et al. (2017). Although real-time LAMP offers operational advantages, being a molecular technique, it necessitates DNA extraction from fecal samples and specialized reaction reagents.

## 5 Conclusion

In UC patients with active disease, high *E. histolytica* infection rates were noted. These findings support considering molecular tests in microscopy-negative cases to improve detection and differentiation of Entamoeba species in high-incidence areas and among high-risk patients. However, cost effectiveness and downstream impact on clinical outcomes warrant further study.

## Data Availability

The datasets presented in this study can be found in online repositories. The names of the repository/repositories and accession number(s) can be found in the article/**Supplementary Material**.
