# Correction: Evaluation of real-time LAMP assay for detection of *Entamoeba* species in clinical stool samples from a tertiary care center in North India

**DOI:** 10.3389/fpara.2026.2017245

**Published:** 2026-10-12

**Authors:** Frontiers Production Office

**Affiliations:** Frontiers Media SA, Lausanne, Switzerland

**Keywords:** *Entamoeba histolytica*, inflammatory bowel disease, molecular diagnostics, real-time LAMP, ulcerative colitis

Affiliation 1 “Department of Medical Parasitology, Post Graduate Institute of Medical Education and Research, Chandigarh, India” was erroneously given as “Department of Parasitology, Institute of Tropical Medicine (NEKKEN), Nagasaki University, Nagasaki, Japan”.

Affiliation 2 “Department of Gastroenterology, Post Graduate Institute of Medical Education and Research, Chandigarh, India” was erroneously given as “The Joint Usage/Research Center on Tropical Disease, Institute of Tropical Medicine (NEKKEN), Nagasaki University, Nagasaki, Japan”.

Affiliation 5 “Department of Parasitology, Institute of Tropical Medicine (NEKKEN), Nagasaki University, Nagasaki, Japan & The Joint Usage/Research Center on Tropical Disease, Institute of Tropical Medicine (NEKKEN), Nagasaki University, Nagasaki, Japan” was erroneously given as “Department of Parasitology, Nagasaki University, Nagasaki, Japan”.

The original version of this article has been updated.

