# Supplementary material for: Evaluation of real-time LAMP assay for detection of Entamoeba species in clinical stool samples from a tertiary care center in North India
Source: Front Parasitol. 2026 Oct 1;5:1937802. doi: 10.3389/fpara.2026.1937802 (PMC13697832; doi:10.3389/fpara.2026.1937802)
Supplement: Supplementary file 1 [file SupplementaryFile1.docx]

|  |  | **Section & Topic** | | **No** | **Item** |  |
| --- | --- | --- | --- | --- | --- | --- |
|  | | |  | | |  |
|  |  | **TITLE OR ABSTRACT** | |  |  | Evaluation of Real-time LAMP assay for detection of Entamoeba Species in Clinical Stool Samples from a Tertiary Care Centre in North India |
|  |  |  | | **1** | Identification as a study of diagnostic accuracy using at least one measure of accuracy  (such as sensitivity, specificity, predictive values, or AUC) | Table 4 |
|  |  | **ABSTRACT** | |  |  |  |
|  |  |  | | **2** | Structured summary of study design, methods, results, and conclusions  (for specific guidance, see STARD for Abstracts) | Abstract section |
|  |  | **INTRODUCTION** | |  |  |  |
|  |  |  | | **3** | Scientific and clinical background, including the intended use and clinical role of the index test | Introduction section |
|  |  |  | | **4** | Study objectives and hypotheses | Final paragraph of Introduction section |
|  |  | **METHODS** | |  |  |  |
|  |  | *Study design* | | **5** | Whether data collection was planned before the index test and reference standard  were performed (prospective study) or after (retrospective study) | Prospective study –Study setting and  study design section. |
|  |  | *Participants* | | **6** | Eligibility criteria | Study subject section. |
|  |  |  | | **7** | On what basis potentially eligible participants were identified  (such as symptoms, results from previous tests, inclusion in registry) | Gastroenterology OPD/Emergency OPD/ Parasitology routine laboratory.  Community recruitment-Study subject section. |
|  |  |  | | **8** | Where and when potentially eligible participants were identified (setting, location and dates) | PGIMER and Manimajra hospital, North India and 3 villages  (Jan 2021-Jan 2023). |
|  |  |  | | **9** | Whether participants formed a consecutive, random or convenience series | Mixed. For patients with UC and non-IBD diarrhea these were enrolled consecutively from the OPD/Emergency Medical OPD; community participants and diseased controls enrolled by convenience sampling. |
|  |  | *Test methods* | | **10a** | Index test, in sufficient detail to allow replication | Real-time LAMP assay section and Table 2 (primer sequences/concentrations). |
|  |  |  | | **10b** | Reference standard, in sufficient detail to allow replication | Conventional PCR and Real-time PCR and Microscopic examination sections. |
|  |  |  | | **11** | Rationale for choosing the reference standard (if alternatives exist) | In the context of the current study, microscopy cannot differentiate among morphologically similar species and PCR is only applicable in well-established setups, therefore Bayesian latent class analysis was used. |
|  |  |  | | **12a** | Definition of and rationale for test positivity cut-offs or result categories  of the index test, distinguishing pre-specified from exploratory |  |
|  |  |  | | **12b** | Definition of and rationale for test positivity cut-offs or result categoriesof the reference standard, distinguishing pre-specified from exploratory | No single reference standard; microscopy, conventional PCR, and real-time PCR were jointly modelled via Bayesian latent class analysis ("Statistical analysis" section) |
|  |  |  | | **13a** | Whether clinical information and reference standard results were available  to the performers/readers of the index test |  |
|  |  |  | | **13b** | Whether clinical information and index test results were available  to the assessors of the reference standard | YES |
|  |  | *Analysis* | | **14** | Methods for estimating or comparing measures of diagnostic accuracy | Bayesian latent class model (105) |
|  |  |  | | **15** | How indeterminate index test or reference standard results were handled | They were reported or excluded from the analyses. |
|  |  |  | | **16** | What missing data on the index test and reference standard were handled | Complete data could not be obtained for 20.15% of UC patients and rest 14% could not be contacted. |
|  |  |  | | **17** | Any analyses of variability in diagnostic accuracy, distinguishing pre-specified from exploratory | Subgroup comparison of Entamoeba positivity in active UC vs remission, not otherwise specified. |
|  |  |  | | **18** | Intended sample size and how it was determined | Study subjects’ section – sample size formula referenced (ref 24). |
|  |  | **RESULTS** | |  |  |  |
|  |  | *Participants* | | **19** | Flow of participants, using a diagram | Figure 1 |
|  |  |  | | **20** | Baseline demographic and clinical characteristics of participants | Study population characteristic section (mean age, sec distribution , UC phenotype and disease duration). |
|  |  |  | | **21a** | Distribution of severity of disease in those with the target condition | UC disease activity (Figure 1 and result section) |
|  |  |  | | **21b** | Distribution of alternative diagnoses in those without the target condition | Diseases controls defined as patients having GI symptoms other than diarrhea. |
|  |  |  | | **22** | Time interval and any clinical interventions between index test and reference standard | All four tests were performed on the same stool sample within 1 week span. Storage and processing of samples and Diagnostic techniques section. |
|  |  | *Test results* | | **23** | Cross tabulation of the index test results (or their distribution)  by the results of the reference standard | Table 3 and Table 4(Dectection rates by method and species) |
|  |  |  | | **24** | Estimates of diagnostic accuracy and their precision (such as 95% confidence intervals) | Table 4. |
|  |  |  | | **25** | Any adverse events from performing the index test or the reference standard | None |
|  |  | **DISCUSSION** | |  |  |  |
|  |  |  | | **26** | Study limitations, including sources of potential bias, statistical uncertainty, and generalisability | Discussion section. |
|  |  |  | | **27** | Implications for practice, including the intended use and clinical role of the index test | Discussion and conclusion section. |
|  |  | **OTHER INFORMATION** | |  |  |  |
|  |  |  | | **28** | Registration number and name of registry | N/A |
|  |  |  | | **29** | Where the full study protocol can be accessed | PGIMER Institutional Ethics Committee approval no. INT/IEC/2022/SPL-766; thesis reference IEC-INT/2022/PhD-314 |
|  |  |  | | **30** | Sources of funding and other support; role of funders | The author(s) declare that no financial support was received for the research and/or publication of this article" – Funding statement |

# STARD 2015

## AIM

STARD stands for “Standards for Reporting Diagnostic accuracy studies”. This list of items was developed to contribute to the completeness and transparency of reporting of diagnostic accuracy studies. Authors can use the list to write informative study reports. Editors and peer-reviewers can use it to evaluate whether the information has been included in manuscripts submitted for publication.

## EXPLANATION

A **diagnostic accuracy study** evaluates the ability of one or more medical tests to correctly classify study participants as having a **target condition.** This can be a disease, a disease stage, response or benefit from therapy, or an event or condition in the future. A medical test can be an imaging procedure, a laboratory test, elements from history and physical examination, a combination of these, or any other method for collecting information about the current health status of a patient.

The test whose accuracy is evaluated is called **index test.** A study can evaluate the accuracy of one or more index tests. Evaluating the ability of a medical test to correctly classify patients is typically done by comparing the distribution of the index test results with those of the **reference standard**. The reference standard is the best available method for establishing the presence or absence of the target condition. An accuracy study can rely on one or more reference standards.

If test results are categorized as either positive or negative, the cross tabulation of the index test results against those of the reference standard can be used to estimate the **sensitivity** of the index test (the proportion of participants *with* the target condition who have a positive index test), and its **specificity** (the proportion *without* the target condition who have a negative index test). From this cross tabulation (sometimes referred to as the contingency or “2x2” table), several other accuracy statistics can be estimated, such as the positive and negative **predictive values** of the test. Confidence intervals around estimates of accuracy can then be calculated to quantify the statistical **precision** of the measurements.

If the index test results can take more than two values, categorization of test results as positive or negative requires a **test positivity cut-off**. When multiple such cut-offs can be defined, authors can report a receiver operating characteristic (ROC) curve which graphically represents the combination of sensitivity and specificity for each possible test positivity cut-off. The **area under the ROC curve** informs in a single numerical value about the overall diagnostic accuracy of the index test.

The **intended use** of a medical test can be diagnosis, screening, staging, monitoring, surveillance, prediction or prognosis. The **clinical role** of a test explains its position relative to existing tests in the clinical pathway. A replacement test, for example, replaces an existing test. A triage test is used before an existing test; an add-on test is used after an existing test.

Besides diagnostic accuracy, several other outcomes and statistics may be relevant in the evaluation of medical tests. Medical tests can also be used to classify patients for purposes other than diagnosis, such as staging or prognosis. The STARD list was not explicitly developed for these other outcomes, statistics, and study types, although most STARD items would still apply.

## DEVELOPMENT

This STARD list was released in 2015. The 30 items were identified by an international expert group of methodologists, researchers, and editors. The guiding principle in the development of STARD was to select items that, when reported, would help readers to judge the potential for bias in the study, to appraise the applicability of the study findings and the validity of conclusions and recommendations. The list represents an update of the first version, which was published in 2003.

More information can be found on [http://www.equator-network.org/reporting-guidelines/stard.](http://www.equator-network.org/reporting-guidelines/stard/)

Reference

1. Bossuyt, P. M. et al. For the STARD Group. STARD 2015: An Updated List of Essential Items for Reporting Diagnostic Accuracy Studies. [cited accessed February 4, 2025].

**2 .**

Analytical sensitivity of (A) Conventional PCR (1ng) (B) Real-time PCR (1pg) (D)Real-time LAMP (10fg)
